# Developing the evidence-base to inform policy on inclusive research design

**DOI:** 10.1098/rsos.241380

**Published:** 2025-03-19

**Authors:** Stella A. Child, Christina Mulligan, Ivan Pavlov, Simone Bryan, Leah Li, Rachel Louise Knowles

**Affiliations:** ^1^ Medical Research Council, Swindon, UK; ^2^ Population, Policy and Practice Research and Teaching Department, UCL Great Ormond St Institute of Child Health, London, UK

**Keywords:** diversity, inclusion, responsible conduct of research, research policy

## Abstract

Considering diversity when designing and conducting research is fundamental to the responsible conduct of research and ensures that outputs from scientific research are reproducible, minimize bias and enable everyone within society the opportunity to benefit. Therefore, health and biomedical research should include consideration of diversity and inclusion in the way studies are designed and conducted. An evaluation of health researchers’ approaches to diversity was undertaken to generate evidence to inform research policy development by the UK Medical Research Council (MRC). Seven hundred and seventy-two researchers responded to an anonymized public survey about diversity and inclusion in research design and 590 applications for research funding were evaluated. Fifty per cent of survey respondents undertaking human participant research reported taking diversity, usually age and sex, into account. Although 43% of animal researchers reported using females and males, only 28% of grant applications demonstrated this. Our findings demonstrate that many researchers do not routinely consider diversity when designing research. Furthermore, we identified a gap between what animal researchers reported doing and what was evident in funding applications. Informed by this analysis, MRC implemented a new policy requiring researchers to demonstrate how they embed diversity and inclusion in research design. This survey provides a benchmark for evaluating policy impact.

## 1. Introduction

There is increasing recognition within health and biomedical research of the need to consider diversity and inclusion in the way in which studies are designed and conducted, for example, in recruiting participants, selecting animal subjects and deciding how analyses are carried out. A key driver for this growing awareness of the importance of ‘inclusive research design’ is the reports of research bias when diversity is ignored. For example, a US Government Accountability Office review in 2001 reported that, despite having regulations requiring proportional representation of sexes in clinical trials in the US since 1993, eight of ten drugs that were withdrawn from the US market by the Federal Drug Administration posed greater health risks to women than men [1]. More recently, the COVID pandemic has had a disproportionate impact on minority ethnic communities, highlighting the importance of a focus on ethnic diversity when undertaking clinical trials into prevention and treatment. Gendered Innovations [2], a Stanford University-based initiative to promote gender and diversity balance in research and innovation, has cited many similar case studies from across different scientific disciplines, including ‘male’ crash test dummies that cannot predict injury in females [3] and facial recognition algorithms that are racially biased [4].

Within preclinical research, concerns have also been raised about sex bias [5,6], failures of translation from animal models into effective clinical applications [7] and a lack of information about donor sex to support the interpretation of cell-based experiments [8,9]. Historically, the reluctance to use female animals was based on concerns about variability caused by the oestrous cycle, however, a meta-analysis has demonstrated that female mice show no more variability than males (and in some cases less) and that there is rarely any need to control for reproductive cycle [10]. Understanding the impact of sex is particularly relevant to clinical therapeutic uses arising from cell-based research, as better outcomes may result from matching the sex of donor and recipient in stem cell transplantation or preferentially using female stem cells over male to treat specific diseases [11–13].

Diversity and inclusion in research design and conduct are attaining greater importance as a dimension of responsible conduct of research, which focuses on the process of how research is carried out, as well as the benefit it brings to wider society [14]. Internationally, research funders have begun developing policies to promote diversity and inclusion in the design and conduct of the studies they fund and to address concerns about the relevance of scientific outputs [15]. The European Commission has encouraged systematic consideration of sex and gender since 2003, and other funders have introduced similar policies on sex, gender and other diversity characteristics [16–19]. These policies cut across scientific disciplines [19]. In the UK, funders have been slower to address this issue, however, the Medical Research Council (MRC) Trials Methodology Hubs and National Institute for Health and Care Research (NIHR) INCLUDE Initiative developed the INCLUDE Ethnicity Framework for randomized trials in 2021 [20]. This led to the NIHR INCLUDE Project, which has produced a range of influential guidance and learning tools to improve the inclusion of underserved groups in clinical trials [21].

The MRC, a major public funder of biomedical and health research and innovation in the UK and part of UK Research and Innovation (UKRI), began developing a research policy on diversity and inclusion in the design of research, involving animal subjects, human participants and cells and tissues in 2021. To ensure that the policy was grounded in evidence, an analysis of funding applications submitted to the MRC was carried out, as well as a survey of the research community about current practice. Information was collected about diversity and inclusion throughout the research lifecycle, from design through participant recruitment or subject selection to the analytical approaches and methods for dissemination of findings. We present here findings from analyses of the survey and grant applications and outline how these have influenced subsequent research policy development and implementation aimed at improving research culture and the responsible conduct of research.

## 2. Material and methods

### 2.1. Analysis of grant applications submitted to MRC

Applications for research grants and fellowships submitted to MRC research funding boards and panels for decision meetings held between April and September 2020 (*n* = 590) were included in the analysis of diversity and inclusion within research proposals, regardless of whether the applications were successful in being awarded funding. Three hundred and seventy-seven grant applications (table 1) involved human participants, or their data were searched for *any mention* of sex, age, ethnicity and/or socio-economic status of research participants in the description of the research proposal. Socio-economic status was broadly defined to include characteristics such as educational attainment, employment or income. If the research proposal *mentioned* one of these diversity characteristics, then any statement explaining their relevance to the study design or how the researchers proposed to include diversity in their analysis was sought. Two hundred and thirty-three applications (table 2) used animal subjects and were searched for information about the sex of the animal to be used and for a statement explaining this choice.

**Table 1.** Research involving human participants: analysis of grant applications and survey responses.

grant applications (*n* = 377)	number of applications	
diversity characteristics addressed		% of 377
diversity characteristic mentioned (at least one)	284	75%
diversity characteristic explained (at least one)	194	51%
analytical approach to diversity explained	69	18%
characteristics considered in design		% of 284
sex	256	90%
age	187	66%
socio-economic position	59	21%
ethnicity	26	9%
characteristics considered in analysis		% of 69
sex	57	83%
age	52	75%
socio-economic position	34	49%
ethnicity	22	32%

applicant gender	number of applications	N (%) mentioning diversity	χ^2^ results
applications by men	219	159 (73%)	χ^2^ = 2.13; *p* = 0.144
applications by women	154	122 (79%)	

awarded applications only (*n* = 56)	number of applications	% of 56	χ^2^ results
diversity characteristic mentioned	44	79%	χ^2^ = 0.37; *p* = 0.542
diversity characteristic explained	24	43%	

survey respondents (*n* = 415; survey questions 2.2 to 2.7)		
diversity considered (at any research stage; survey question 2.2)		
always	207	50%
sometimes	148	36%
rarely	37	9%
never	16	4%
not answered	7	2%

when diversity was considered by respondents who always considered diversity (*n* = 207) (survey questions 2.3−2.6)	always	sometimes	rarely	never	not answered
in recruitment (question 2.3)	111 (54%)	77 (37%)	7 (3%)	4 (2%)	8 (4%)
in methods (question 2.4)	94 (45%)	86 (42%)	9 (4%)	12 (6%)	6 (3%)
in analysis (question 2.5)	120 (58%)	71 (34%)	3 (1%)	6 (3%)	7 (4%)
in reporting (question 2.6)	108 (52%)	84 (41%)	4 (2%)	4 (2%	7 (3%)

diversity characteristics considered by respondents who always considered diversity (*n* = 207) (survey question 2.7)^a^	number of respondents	% of 207*
age	200	97%
sex/gender	196	95%
ethnicity/race	155	75%
socio-economic position	112	54%
disability	49	24%

This table presents an overview of the 377 grant applications that included human participant research and the survey responses provided by 392 respondents who undertook research with human participants. The diversity characteristics listed in relation to the grant applications (sex, age, socio-economic position and ethnicity) are those that were searched for within applications.

^a^ This part of the table only presents the survey responses for the 207 survey respondents who said they considered diversity *always*; we have excluded those who *sometimes*, *rarely* or *never* considered diversity or did not answer.

**Table 2.** Research involving animals: analysis of grant applications and survey responses.

grant applications (total *n* = 233)	number of applications	
sex specified (*n* = 118)		% of 233
sex specified (male, female, both)	103	44%
will use all offspring of breeding programme (regardless of sex)	15	6%
of 103 applications not using all offspring:		% of 103
both sexes	65	63%
female only	20	19%
male only	18	17%

differences by gender (*n* = 118*)	number of applications	*N* (%) using both sexes	*χ*^2^ results
applications by men	74	51 (69%)	*χ*^2^=1.09; *p* = 0.581
applications by women	44	28 (64%)	

differences by application success (*n* = 118^a^)	number of applications	*N* (%) using both sexes	*χ*^2^ results
awarded applications	15	11 (73%)	*χ*^2^=0.41; *p* = 0.813
declined applications	103	68 (66%)	

survey responses (*n* = 240)	number of respondents	
respondents' approach to sex in the experimental design of animal studies (survey question 3.3)		% of 240
use equal numbers of males and females	103	43%
use both sexes if relevant to the research question	68	28%
use both sexes to compare outcomes in males and females	60	25%
carry out a pilot study to test for sex differences and, if there are none, run the main experiment with one sex	21	9%
use equal numbers of males and females only if there is a literature precedent	15	6%
runs experiments in duplicate, in males then in females	10	4%
use one sex in all (or most) experiments	75	31%
respondents’ approach to analysis and reporting by sex (survey question 3.6)		% of 240
reports the sex of the animals used in publications	166	69%
includes sex as a factor in the analysis	80	33%
only reports analyses by sex if a sex difference is found	47	20%
reports the results separately for each sex	39	16%
does not analyse or report by sex as only one sex used	31	13%
does not analyse or report by sex although both sexes used	20	8%

This table presents an overview of the 233 grant applications that included animal research and the survey responses provided by 240 respondents who undertook animal research.

^a^ 118 applications specified the sex of the animals to be used or indicated all offspring would be used.

### 2.2. Survey

The cross-sectional online survey was developed by MRC policy staff and comprised six sections, each with 3–17 questions. As there were no existing UK research funder policies on inclusive research design, the survey was primarily designed to establish whether researchers reported considering diversity in any way, with further questions to understand *how* diversity was taken into account in analyses and to explore researchers’ views of the benefits and drawbacks of inclusive research design. Three of the sections covered awareness of existing diversity guidance, attitudes towards diversity in public involvement, and the role that funders should play in promoting inclusive research design. Three further sections were specifically targeted at research involving (a) animals, (b) human participants and (c) cell and tissue samples (electronic supplementary material, table S1). The online survey included multiple-choice questions, routing, pre-programmed answer fields (e.g. number, text, date) and restrictions on the length of free text to minimize entry errors.

An email invitation to complete the survey was extended to all MRC lead applicants of active or recently completed (within 5 years) funding awards, as well as staff at MRC-funded units, centres, institutes and research organizations receiving MRC funding. To ensure a broad range of responses from throughout the research community, the survey link was not restricted to MRC-funded researchers and was distributed to other UK funders, NHS research contacts, and other relevant organizations. The survey link was also disseminated via MRC newsletters and social media. The survey was open for one month, from 8 July to 8 August 2021, and data were held on a secure server. Respondents completed the survey anonymously, therefore, we could not implement any procedures to detect whether individuals responded more than once.

**Figure 1.**
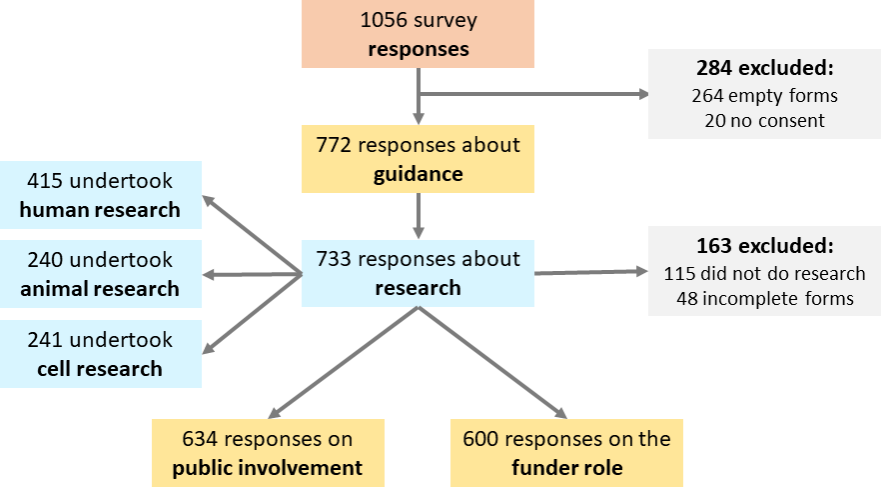
Survey responses and exclusions

Of 1056 responses received, 284 were excluded because they were incomplete or the respondent had not consented to the processing of their data, thus 772 responses were included. In the analyses presented below, the denominator used is the number of respondents who completed the relevant section (figure 1).

### 2.3. Statistical analyses

Results are presented as counts and percentages. Chi-squared tests were used to explore relationships between respondent characteristics and the use of male and female animals or consideration of diversity in humans. Odds ratios (ORs) and 95% confidence intervals (95% CI) were estimated using logistic regression to determine whether any survey respondent characteristics influenced the use of female and male animals or consideration of diversity in humans. Analyses were undertaken in Stata 18.

## 3. Results

### 3.1. Grant applications

#### 3.1.1. Human research applications

Of 377 applications involving human participants, 284 (75%) reported at least one diversity characteristic in the description of their research proposal, and 194 (51%) explained *why* they had chosen this characteristic or how they planned to use it in the analysis (table 1). Sex was most likely to be mentioned (*n* = 256), followed by age (*n* = 187), while socio-economic position (*n* = 26) and ethnicity (*n* = 59) were discussed in fewer than one-quarter of applications. Only 69 (18%) applications described how sex or other diversity characteristics would be managed in the analysis.

Of 56 applications that were *awarded* funding, 44 (79%) mentioned at least one diversity characteristic but only 24 (43%) provided an explanation for this choice (table 1). Grant applications that mentioned diversity did not appear to be more successful than those that did not (*χ*^2^ = 0.37; *p* = 0.542) and the gender of the grant applicant was not associated with the likelihood of mentioning a diversity characteristic (table 1; *χ*^2^ = 2.13; *p* = 0.144).

#### 3.1.2. Animal research applications

Of 233 applications involving animals (table 2), 118 stated the sex to be used. Of these, 15 (6%) planned to use all offspring from their in-house breeding programmes while 103 (44%) specified the sex of the animals: 65 planned to use female and male animals, and 38 planned to use one sex, with similar numbers preferring male and female animals (*n* = 20 female, *n* = 18 male).

Grant applications that used both sexes did not appear to be more successful than those that used one sex only (table 2; *χ*^2^ = 0.41; *p* = 0.813), suggesting this was not a critical factor for those making the funding decision. The use of female and male animals in a study did not appear to be associated with the gender of the grant applicant (table 2; *χ*^2^ = 1.09; *p* = 0.581).

### 3.2. Survey results

#### 3.2.1. Survey responses

Most survey respondents (*n* = 589) completed the demographic details section (table 3). Of these, 324 (55%) were women, 427 (72%) were of White/White British ethnicity and 40 (7%) had a disability. Most were aged 30−60 years (similar frequency for 30−44 years *n* = 214 [36%] and 45−60 years *n* = 218 [37%]). The majority were based in a university (*n* = 497; 84%). Survey respondents selected a career stage from a pre-specified list (heads of department; principal researchers; early career researchers; postdoctoral researchers; PhD students; other). For comparisons between career stages, we derived three groups of respondents (senior level; mid-/early career level; training level).

**Table 3.** Characteristics of survey respondents.

characteristic		number	% of 589^a^
gender	women	324	55%
(survey question 7.5)	men	230	39%
	non-binary/other	11	2%
	not stated (or missing data)	24	4%
age group	18−29 years	64	11%
(survey question 7.6)	30−44 years	214	36%
	45−60 years	218	37%
	over 60 years	75	13%
	not stated (or missing data)	18	3%
ethnic group	Asian/Asian British	46	8%
(survey question 7.7)	black/black British	27	5%
	mixed/multiple ethnic groups	29	5%
	other ethnic group	15	3%
	white/white British	427	72%
	not stated (or missing data)	45	8%
disability	yes	40	7%
(survey question 7.8)	no	528	90%
	not stated (or missing data)	21	4%
type of organisation	university	497	84%
(survey question 7.2)	NHS/health provider	37	6%
	charity/non-governmental organisations	21	4%
	contract research organisation	10	2%
	industry	5	1%
	other	18	3%
	not stated (or missing data)	1	<1%
career level/job title^b^	head of department/senior level	41	7%
(survey question 7.1)	principal investigator/researcher	293	50%
	early career researcher	64	11%
	postdoctoral researcher	53	9%
	PhD student	66	11%
	trial manager/co-ordinator	33	6%
	other (e.g. technician, project manager)	35	6%
	not stated (or missing data)	4	<1%
full-time or not	full time	513	87%
(survey question 7.4)	less than full time	75	13%
	not stated	1	<1%

This table presents respondent characteristics.

^a^ Only 589 of 772 survey respondents provided demographic data.

^b^ To compare career stages, we created three groups (*senior* = Head of department/senior researcher; *early/mid-career* = principal investigator/researcher, early career researcher, postdoctoral researcher, trial managers/co-ordinators; *training* = PhD students) and then excluded the small ‘Other’ group as they were a heterogeneous and poorly defined category.

#### 3.2.2. Awareness of existing guidance on diversity and inclusion

One-third (*n* = 244; 32%) of 772 survey respondents reported that they were *‘aware of guidance on diversity and inclusion in research’* (table 4). When asked to name specific examples, these included guidance on diversity in research teams and organizations, on recruiting research participants or public involvement partners and selecting animal subjects, as well as relating to experimental design approaches (electronic supplementary material, table S2). A higher percentage of senior researchers and heads of department were aware of guidance compared with those at earlier stages of their career: 54% at senior level, 36% at mid-/early career level and 15% at training level, respectively (table 5).

**Table 4.** Respondents’ knowledge of existing guidance on diversity and inclusion in research design.

awareness of guidance (survey question 1.1)	number	% of 772
aware of guidance on diversity	244	32%
not aware of guidance on diversity	523	68%
not answered	5	<1%

awareness of guidance by career stage (survey questions 1.1, 7.1)	number	% of category
senior (*n* = 41)	22	54%
mid-/early career (*n* = 443)	158	36%
training (*n* = 66)	10	15%
other (*n* = 35)	10	29%
not stated (*n* = 187)	44	24%

This table presents respondents’ answers to a survey question asking if they knew of guidance about diversity and inclusion in research design; results are presented for all survey respondents and by respondent career stage.

Career stages were grouped into the following: (i)Heads of department; principal researchers = senior. (ii) Early career researchers; postdoctoral researchers = mid-/early career. (iii) Training = PhD students.(iv) Other = any respondent who did not fit into the above categories.

**Table 5.** Perceived role of funders in influencing diversity in research proposals (*n* = 600 respondents).

funders should develop research funding policy to influence:		
	diversity in the design of research (survey question 6.1)	diversity in research teams (survey question 6.2)
very important	347	242
somewhat important	225	260
not important	22	88
not answered (blank)	6	10

funders should influence diversity in the design of research (survey questions 6.1, 7.5−7.7; *n* = 594^a^ responses)			
	number who responded	*N* (%) stating that influencing diversity in research design is very or somewhat important (compared with not important)	*χ*^2^ results
gender	women (*n* = 322)	317 (98%)	*χ*^2^ = 8.549; *p* = 0.003
	men (*n* = 226)	212 (94%)	
age group	18−44 years (*n* = 276)	268 (97%)	*χ*^2^ =0.358; *p* = 0.550
	45+years (*n* = 289)	278 (96%)	
ethnic group	white/white British (*n* = 422)	408 (97%)	*χ*^2^ = 2.024; *p* = 0.155
	other ethnic groups (*n* = 116)	115 (99%)	

funders should influence diversity in the research team (survey questions 6.2, 7.5−7.7; *n* = 590^a^ responses)			
	number who responded	N (%) stating that influencing research team diversity is very or somewhat important (compared with not important)	*χ*^2^ results
gender	women (*n* = 320)	289 (90%)	*χ*^2^ = 10.695; *p* < 0.001
	men (*n* = 226)	182 (81%)	
age group	18−44 years (*n* = 277)	248 (90%)	*χ*^2^ = 5.235; *p* = 0.022
	45+years (*n* = 286)	237 (83%)	
ethnic group	white/white British (*n* = 422)	360 (85%)	*χ*^2^ = 1.940; *p* = 0.164
	other ethnic groups (*n* = 114)	103 (90%)	

For 600 survey respondents who undertook all types of research, this table presents their views on how important it is for funders to enact policies about (i) increasing diversity in research design and conduct, and (ii) ensuring research is carried out by diverse teams. The variation in responses to these questions by respondent gender, age and ethnicity is shown.

^a^ Denominator excludes ‘Not answered (blank)’ responses, i.e. 6 of 600 respondents did not answer the question about diversity in the design of research, and 10 did not answer the questions about diverse research teams.

#### 3.2.3. Role of funders in promoting diversity

Of 772 respondents to the survey, 639 (83%) considered that the primary responsibility for developing and disseminating guidance about diversity and inclusion in research design lay with research organizations, while 550 (71%) suggested it was funders and 543 (70%) research regulatory bodies (electronic supplementary material, table S3).

Six hundred survey respondents answered specific questions about the role of funders, of whom 572 (95%) thought it was important for funders to have a role in influencing diversity in research design and 502 (84%) considered it important for funders to influence the diversity of research teams (table 5). Women were significantly more likely to consider funder influence on diversity in research design very or somewhat important compared with men (table 5; 98%, 94%, respectively; *χ*^2^ = 8.549, *p* = 0.003) but there was no significant difference in response by age group or ethnicity (table 5; age group 18−44 years versus 45+ years *χ*^2^ = 0.358; *p* = 0.550; ethnicity White/White British versus all other *χ*^2^ = 2.024; *p* = 0.155). To support applicants and peer reviewers, respondents also favoured written guidance on inclusive research design or a joint approach of guidance and training over training alone (electronic supplementary material, table S4).

Most respondents (84%) also indicated that it was important for funders to consider the diversity of the research team when funding research proposals (table 5). Women were significantly more likely to consider funder influence on research team diversity very or somewhat important compared with men (90% and 80%, respectively; *χ*^2^ = 10.695, *p* < 0.001), as were respondents aged under 45 years compared with those who were 45 years or older (table 5; age group 18−44 years versus 45+ years *χ*^2^ = 5.235; *p* = 0.022) but there were no significant differences by ethnic group (table 5; ethnicity White/White British versus all other *χ*^2^ = 1.940; *p* = 0.164).

#### 3.2.4. Diversity in public involvement and engagement

Of 634 respondents who responded to a question about public involvement and engagement (PIE), 206 (32%) always involved patients or the public, 227 (36%) sometimes, 37 (6%) rarely and 23 (4%) never undertook PIE or stated that it was not applicable to their research. Of those who answered this question, 462 (73%) attempted to include diverse or underserved groups (electronic supplementary material, table S5).

#### 3.2.5. Respondents who undertook research

We excluded 163 respondents who did not undertake human, animal or cell research, thus 570 answered questions about their research.

#### 3.2.6. Research involving human participants and data

There were 415 respondents who undertook research involving human participants and/or their data (figure 1). Half (*n* = 207; 50%) *always* considered diversity in their research, while 148 (36%) sometimes, 37 (9%) rarely and 16 (4%) never considered it (table 1).

Of 207 respondents who reported always considering diversity*,* 200 (97%) respondents considered age, 196 (95%) sex/gender, 155 (75%) ethnicity/race, 112 (54%) socio-economic position and 49 (24%) disability (table 1). These respondents also considered diversity at different stages of the research lifecycle: 111 (54%) always considered diversity when recruiting participants, 94 (45%) always adjusted their methods, and 120 (58%) always adjusted their analyses to account for diversity, and 108 (52%) always reported their results against different diversity characteristics.

The respondent characteristics of gender, age and career stage were not associated with whether respondents *always* considered diversity (electronic supplementary material, table S8A).

#### 3.2.7. Research involving animals

Of 240 survey respondents who carried out animal research, there were 124 whose research also involved human participants (table 2).

Male and female animals were used in research undertaken by 103 (43%) respondents, and the choice not to use both female and male animals was influenced by factors such as the research question, pilot studies and precedents in the literature (table 2). Most researchers (*n* = 166; 69%) reported the sex of the animals they used in their papers, but a substantial minority (*n* = 47; 20%) only did so if they found a sex difference. Only 80 (33%) researchers routinely included sex as a factor in their analysis and 20 (8%) did not analyse or report their findings by sex despite using males and females in their experiments.

The respondent characteristics of gender, age and career stage were not associated with whether respondents used female and male animals or only one sex (electronic supplementary material, table S8B).

Researchers who used only one sex of animal (*n* = 75) did so (i) *‘to reduce animal numbers’* (*n* = 18) or (ii) because they were *‘researching a disease or mechanism present in only one sex’* (*n* = 15; table 6). They were as likely to use females as males (females *n* = 27; males *n* = 22; not specified *n* = 26).

**Table 6.** Survey respondents’ reasons for only using one sex of animals (*n* = 75).

reasons given^a^ (survey question 3.4)	number of respondents	% of 75
to reduce animal numbers	18	24%
researching a disease/mechanism present in one sex only	15	20%
to reduce costs	11	15%
to keep the analysis simple	9	12%
to avoid variability due to reproductive cycles	8	11%
there is a literature precedent to use only one sex	7	9%
preferred model only suitable in one sex	7	9%
assumption that sex will have negligible effect	6	8%

For 75 survey respondents who undertook research with animals and used only one sex of animal, this table shows the reasons why they chose to do this and the percentage of respondents who selected each reason.

^a^ Respondents selected reasons from a predefined list or chose ‘Other’ (table 4). Respondents could select more than one reason.

Respondents also reported how they manage other aspects of animal diversity, such as the life stage and the female reproductive or oestrus cycle (electronic supplementary material, tables S6 and S7). About half always used adult animals (*n* = 116; 48%). The most common reason for using animals of a life stage other than adult was because it was *‘relevant to the research question’* (*n* = 100; 42%) and a third based their choice of life stage on when the phenotype was expressed (*n* = 79; 33%).

Many respondents using female animals did not consider reproductive cycle in their experimental design (*n* = 91; 38%). Some respondents stated that it was not important (*n* = 38; 16%), and others were concerned about adding logistic complexity (*n* = 59; 25%). A small number of researchers (*n* = 20; 8%) restricted their experiments to male animals to avoid needing to control for the reproductive cycle (electronic supplementary material, table S7).

#### 3.2.8. *In vitro* studies using cells and tissues

Of 241 respondents who undertook *in vitro* studies using cells and tissues, 92 (38%) only used human cells, 19 (8%) only used animal cells, and 130 (54%) used both human and animal cells (table 7). One-quarter of respondents (*n* = 60; 25%) did not take account of cell donor diversity in their research.

**Table 7.** *In vitro* research using cells and tissues (survey responses).

			number of respondents			% of 241
sources of cells and tissues used by respondents (survey question 4.1)						
human only			92			38%
animal only			19			8%
human and animal			130			54%
researchers: (survey questions 4.2−4.5)	always	sometimes	rarely	never	not answered	
consider cell donor sex/diversity in their experimental design	49	65	47	60	20	
include donor sex/diversity in the analysis	39	64	52	65	21	
report donor sex/diversity characteristics in publications	54	62	37	67	21	
report results by sex/diversity characteristics	43	71	39	66	22	
diversity characteristics taken into account by 161 respondents who always, sometimes or rarely consider diversity when undertaking *in vitro* research; survey question 4.7)			number of respondents			% of 161
donor sex			107			66%
donor age (or life stage)			100			62%
donor race/ethnicity (humans)			56			35%
donor strain/genotype (animals)			78			48%

This table describes survey responses by 241 researchers who undertook *in vitro* research using cells and tissues, including (i) whether they used human or animal cells and tissues, (ii) their views on the importance of considering diversity (represented by the sex, age and ethnicity/genotype of the cell donor) at different stages of the research lifecycle and (iii) which characteristics they were most likely to consider.

Of 161 respondents who considered diversity *‘always, sometimes or rarely’* when designing *in vitro* experiments (table 7), 107 (66%) considered donor sex, 100 (62%) donor age, 56 (35%) donor race/ethnicity (in humans) and 78 (48%) donor strain/genotype (in animals).

#### 3.2.9. Benefits and barriers to considering diversity in research design

Respondents undertaking research with human participants highlighted the key benefits of taking account of diversity (table 8) as an increased likelihood of their findings being relevant (85%), avoiding bias (81%), improving reproducibility (41%), and generating novel findings or hypotheses (31%). Researchers who undertook research with animals or cells reported and ranked the benefits in a very similar way (table 8). A minority of respondents claimed there were no benefits to considering diversity in research, however, many noted important barriers, including increased complexity of the study design (mentioned by 47% in research with human participants, 43% for animal research and 54% for cell research), increased research costs (40%, 56%, 37%, respectively) and greater complexity of analyses (30%, 22%, 9%, respectively). Difficulties in recruiting participants were a main concern for over half of researchers working with humans, while the increased cost of animals and their care and difficulty with obtaining diversity information for cell or tissue samples sourced from commercial suppliers were of greater concern for animal and cell researchers, respectively.

**Table 8.** Benefits and barriers to taking account of diversity in the design and conduct of research.

	respondents – human research (*n* = 415)	respondents – animal research^b^ (*n* = 240)	respondents – cell research (*n* = 241)
benefits	survey question 2.8	survey question 3.7	survey question 4.8
more likely to detect sex-specific differences	—	139 (58%)	—
findings are more likely to be relevant	354 (85%)	114 (47%)	—
avoidance of bias	337 (81%)	133 (55%)	—
increases translation to clinical research	—	95 (40%)	134 (56%)
increased reproducibility of results	172 (41%)	92 (38%)	132 (55%)
more likely to generate novel hypotheses/findings	129 (31%)	59 (25%)	113 (47%)
increased interest in my results	115 (28%)	36 (15%)	52 (22%)
easier to recruit participants	36 (9%)	—	—
easier to publish findings	33 (8%)	23 (10%)	13 (5%)
easier to get ethics or Home Office approval	—	24 (10%)	—
representative of the real world^a^	15 (4%)	—	—
addresses social justice issues^a^	13 (3%)		
exploring diversity is relevant to my research topic^b^	12 (3%)		
no benefits	6 (1%)	—	—
explores genetic differences that are important^b^	3 (<1%)		

drawbacks and barriers	survey question 2.9	survey question 3.8	survey question 4.9
harder to recruit participants	231 (56%)	—	—
concerns that using a larger number of animals would conflict with 3Rs principles	—	111 (46%)	—
more complex study design	193 (47%)	103 (43%)	129 (54%)
increased cost of studies	164 (40%)	134 (56%)	89 (37%)
more difficult to analyse	125 (30%)	54 (22%)	21 (9%)
harder to source biological samples or tissues	109 (26%)	—	117 (49%)
harder to compare outputs to previous studies/deviates from published precedents	105 (25%)	46 (19%)	22 (9%)
preferred model only suited to single sex	—	34 (14%)	—
decreased reproducibility of results	29 (7%)	23 (10%)	—
no drawbacks	41 (10%)	16 (7%)	41 (17%)

This table lists the benefits and barriers to taking account of diversity in research that survey respondents identified. The benefits and barriers associated with human, animal and *in vitro* research are compared in the three columns and listed in order from most frequently to least frequently mentioned by respondents.

^a^ For animal research, separate questions were asked about the benefits and barriers to including sex in the design and analysis of research. Results are shown for the questions about sex but not age.

^b^ These benefits were not part of the original list of options in the survey but were suggested by >1 respondent under ‘Other benefits’.

## 4. Discussion

Around half of those carrying out research with human participants reported *always* taking diversity into account, and this was supported by an analysis of MRC grant applications, which mentioned diversity characteristics in 75% of proposed research. Age and sex were the diversity characteristics most likely to be included in the study proposal, with ethnicity, socio-economic position and disability less often mentioned. Among those doing animal research, although 43% of researchers reported using males and females in their studies, only 28% discussed this in grant applications. Overall, our analysis suggested that diversity is often not explicitly considered in the design of research proposals. Survey respondents expressed concerns about the potential increased costs of inclusive research design. A high percentage (72%) of survey respondents stated that funders should take responsibility for providing written guidance on including diversity in research design. Taken together, this evidence underlines the need for funders to use policy and guidance effectively to promote inclusive approaches to the design and conduct of health and biomedical research, to enhance reproducibility, avoid bias and ensure research outputs are relevant to social needs.

### 4.1. Comparison with previous studies

Policies on inclusive research design have been introduced and evaluated by other funders, including in Canada and the US. However, we identified only a limited number of previous studies that have evaluated diversity within grant applications or assessed researcher views of inclusive research practice; these reported results were largely consistent with our findings. One survey, undertaken two years after the implementation of the US National Institutes of Health (NIH) ‘Sex as a Biological Variable’ (SABV) in 2015, collected the views of members of NIH funding panels (study-sections) [22]. The authors noted that 76% of researchers always reported sex in their publications, which was similar to our survey findings for researchers using animals (69%), although the studies are not directly comparable as the US study was undertaken after, and our study before, policy implementation. The key benefits of an inclusive research design identified in the US survey were improved translation and reproducibility, while key concerns were potential increases in research costs and an increase in the number of animals used. In our survey, a potential increase in the number of animals required was also frequently raised as a concern despite good evidence that optimising statistical methods can avoid this [6]. Researchers in our survey were also apprehensive about the potential increase in research costs, suggesting this is an important issue that funders should address in their applicant guidance.

Two years after the implementation of the NIH SABV policy, Freeman and co-authors assessed the impact on study proposals involving human participation within one research organization [23]. Although 69% of NIH applicants mentioned the sex/gender of participants, only 14% explained their choice and 2% proposed to analyse sex differences. This contrasts with our findings that, without a policy in place, 50% of MRC applications explained the choice of at least one diversity characteristic. Our findings may differ due to the types of applications reviewed, the role of the host institution, or reflect a marked increase in awareness of the importance of considering sex and gender over recent years.

Various authors have reported that research teams led by women are significantly more likely to take account of diversity, including analysing data by sex or reporting the sex of animals in their publications [24–26], however, we identified no significant difference by gender of the research team lead. Among our survey respondents, women were significantly more likely to consider it important for funders to promote diversity in research design compared with men.

The Canadian Institutes for Health Research (CIHR) implemented a policy on sex and gender in 2010 and included new questions in application forms that asked applicants to confirm they had addressed these issues. Between 2010 and 2012, the percentage of applicants confirming they had addressed sex/gender increased from 26% to 48%, suggesting a significant positive impact of the new policy [27]. A qualitative study of the NIH SABV policy also reported a small non-significant increase from 2016 to 2017 in the percentage of researchers who considered a sex and gender policy important and thought that it would improve reproducibility [22].

### 4.2. Strengths and limitations

We received a large number of responses to the survey, and although most respondents were from UK universities, they were diverse in terms of gender, age, ethnic group and career stage. As a group, respondents were representative of the researchers who receive MRC funding and will be influenced by future policy. As we did not know how many people had received the survey link, we could not calculate the response rate. The survey was completed anonymously therefore it was possible for an individual to complete the survey more than once.

The survey had several sections but not all were completed by, or relevant to, every respondent, which resulted in a varying proportion of missing data within each section. Nevertheless, the overall completion rate was good, with several hundred responses to each section. We relied on self-completion of demographic information to enable us to draw comparisons between responses from individuals by gender, age and career stage, thus missing data in this section may have limited our ability to identify significant differences between respondent subgroups.

The survey was short in order to optimize completion rates and focused on capturing whether researchers demonstrated a basic level of awareness of diversity and inclusive research practice. A limitation of this approach was that the survey did not explore the different ways in which diversity was taken into account by researchers, from inclusive recruitment to analytic approaches. This survey, therefore, provides a foundation for understanding researcher perspectives on diversity and inclusive research, although further work is needed to fully understand how inclusive approaches are used within current research practice.

The analysis of MRC grants only included applications considered for funding between April and September 2020 however we included applications to all MRC research boards and panels and thus analysed a varied range of studies from basic discovery, preclinical and translational studies to data science, clinical and population health research. Funding rounds from this period were selected as they were the most recent rounds from each funding opportunity that had complete information about the funding decision at the time of analysis. These included training fellowship applications from junior applicants. Our analysis of grant proposals was limited to the information that was available in application forms, so it primarily reflects explicit mention of diversity. Various applicants explained why specific characteristics were relevant to their study proposal and how they would ensure inclusive recruitment or referred to analysis and reporting, however, the detail provided was limited to what was required for the application process therefore insufficient for us to explore different approaches to analysis. It is possible that more applicants took diversity into account but did not state this in their application as they did not consider it relevant or did not expect to be assessed on this aspect.

## 5. Conclusion

Embedding diversity and inclusion in the design and conduct of research enhances the quality of scientific research, improving the experimental design, making research more relevant to the needs of society, increasing opportunities for new discoveries and reducing the likelihood of bias [28]. Inclusive research design is part of the wider framework of the responsible conduct of research and crucial to enhancing public trust in scientific aims and outputs.

As Hunt *et al.* have highlighted, *'funding agencies are one of the three pillars of the science infrastructure that need to coordinate [diversity and inclusion] policies to achieve excellence in science’* [15]. It is therefore an important responsibility of funders to address inequity and bias [29], and to promote scientific rigour and reproducibility, through implementing research funding policy and strategy that nurtures inclusive research practice.

Following recommendations from an expert working group who considered the evaluation reported in this paper alongside other evidence, the MRC produced guidance on the use of both sexes in animal research in 2022 [30] and launched a new policy on *Embedding Diversity in Research Design* in 2023. The findings reported in this paper have established a benchmark against which changes to research culture and practice in the light of the new MRC research policy will be evaluated.

## Data Availability

Individual-level data from the survey cannot be provided as this could be disclosive of the identity of respondents or their institutions. The article and supplementary files include aggregated tables which contain all of the data to support the findings reported. The original dataset is held in the MRC Science Archive and can be requested by application to the MRC Data Access Committee [31]. Child, Stella; Mulligan, Christina; Pavlov, Ivan; Bryan, Simone; Li, Leah; Knowles, Rachel (2025). CROSS checklist: Developing the evidence base to inform policy on inclusive research design.docx. figshare. Online resource [32]. Supplementary material is available online [33].
